# Comparison of Phytochemical Profiles of Wild and Cultivated American Ginseng Using Metabolomics by Ultra-High Performance Liquid Chromatography-High-Resolution Mass Spectrometry

**DOI:** 10.3390/molecules28010009

**Published:** 2022-12-20

**Authors:** Zhihao Liu, Roderick Moore, Ying Gao, Pei Chen, Liangli Yu, Mengliang Zhang, Jianghao Sun

**Affiliations:** 1 Methods and Application of Food Composition Laboratory, Beltsville Human Nutrition Research Center, Agricultural Research Service, United States Department of Agriculture, Beltsville, MD 20705, USA; 2 Department of Nutrition and Food Science, University of Maryland, College Park, MD 20742, USA; 3 Department of Chemistry, Middle Tennessee State University, Murfreesboro, TN 37132, USA; 4 School of Agriculture, Middle Tennessee State University, Murfreesboro, TN 37132, USA

**Keywords:** American ginseng, wild, cultivated, ginsenoside, discrimination, HRMS

## Abstract

American ginseng (*Panax quinquefolius* L.) has been recognized as a valuable herb medicine, and ginsenosides are the most important components responsible for the health-beneficial effects. This study investigated the secondary metabolites responsible for the differentiation of wild and cultivated American ginsengs with ultrahigh-performance liquid chromatography-high resolution mass spectrometry (UHPLC-HRMS)-based metabolomic approach. An in-house ginsenoside library was developed to facilitate data processing and metabolite identification. Data visualization methods, such as heatmaps and volcano plots, were utilized to extract discriminated ion features. The results suggested that the ginsenoside profiles of wild and cultivated ginsengs were significantly different. The octillol (OT)-type ginsenosides were present in greater abundance and diversity in wild American ginsengs; however, a wider distribution of the protopanaxadiol (PPD)-and oleanolic acid (OA)-type ginsenosides were found in cultivated American ginseng. Based on the tentative identification and semi-quantification, the amounts of five ginsenosides (i.e., notoginsenoside H, glucoginsenoside Rf, notoginsenoside R1, pseudoginsenoside RT2, and ginsenoside Rc) were 2.3–54.5 fold greater in wild ginseng in comparison to those in their cultivated counterparts, and the content of six ginsenosides (chicusetsusaponin IVa, malonylginsenoside Rd, pseudoginsenoside Rc1, malonylfloralginsenoside Rd6, Ginsenoside Rd, and malonylginsenoside Rb1) was 2.6–14.4 fold greater in cultivated ginseng compared to wild ginseng. The results suggested that the in-house metabolite library can significantly reduce the complexity of the data processing for ginseng samples, and UHPLC-HRMS is effective and robust for identifying characteristic components (marker compounds) for distinguishing wild and cultivated American ginseng.

## 1. Introduction

American ginseng (*Panax quinquefolius* L.) is one of the most popular herbal medicines in the world and is known for its diverse pharmacological activities [1]. The roots are used as dietary health supplements and additives to food, beverages, and cosmetics and for the treatment of many human ailments, such as fatigue, neurodegradation, cardiovascular diseases, stress, and cancer [2,3]. Several groups of bioactive substances, such as saponins, polysaccharides, alkaloids, polyacetylenes, and phenolic acids, are associated with their diverse health-promoting properties [4]. The saponins from ginseng, also known as ginsenosides, are considered the major bioactive constituents of ginseng with many reported bioactive effects such as antidiabetic activity, prevention of tumors, and subsidiary treatment of coronary heart disease and chronic hepatitis [5]. Over 300 different ginsenosides have been reported, and most of them can be classified into four groups based on their molecular structures [6]. As shown in Figure S1, these classes include protopanaxadiol (PPD), protopanaxatriol (PPT), oleanolic acid (OA), and ocotillol (OT) type ginsenosides, and their presence varied significantly between different ginseng species. For example, the OT-type ginsenoside F11 is exclusively found in American ginseng and has previously been used to confirm the identity of American ginseng, while the PPT-type ginsenoside Rf is used to verify the identity of Korean ginseng [5]. Similarly, levels of the PPD-type ginsenoside Rb1, were significantly varied between wild-simulated and woods-cultivated American ginseng [7]. Biochemical and pharmacological studies on ginsengs have mainly concentrated on ginsenosides. Other chemical constituents in ginseng, including polyacetylenes, phenolic compounds (flavonoids and phenolic acids), essential oils, polysaccharides, microelements, and vitamins, are also responsible for the complex pharmacological activities of ginseng [8,9].

American ginseng is widely cultivated in North America, and wild ginseng can be found in the eastern and central states of the United States and Southeastern Canada. In addition to its medical properties, American ginseng is a cash crop and export of North America, with samples harvested in the wild going for 10-fold the cost of cultivated roots [7]. Overharvesting wild American ginseng is a major concern due to its popularity, unique habitat requirements, and rarity [10]. Wild American ginseng has been listed in Appendix II of the Convention on the International Trade in Endangered Species of Wild Fauna and Flora (CITES) since 1974, and its harvest and trade are strictly regulated by the US Fish and Wildlife Service (USFWS) [11]. Due to the scarcity of wild-harvested ginseng, there was limited research on the wild populations of American ginseng. A phytochemical study showed the ginsenosides in wild American ginsengs from ten populations in both US and Canada varied from 1 to 16% [12]. The wild ginseng from the Eastern US has high ginsenoside Rg1 and low ginsenoside Re, which is distinctive from that of the cultivated ginseng as well as from the wild ginseng grown in the Northern US and Canada. In a recent study, we reported that Tennessee wild ginseng had significantly higher ginsenoside contents and more ginsenoside diversity than cultivated ginseng. Marked chemotypic differences between the wild and cultivated ginseng were also reported, such as ratios of PPD/PPT, ginsenoside Rg1/Rb1, Rg1/Re, and Rb2/Rc [13].

The growth of ginseng is greatly influenced by many factors, including germplasm, geographical origin, cultivation environment, harvesting, storage, postharvest processing, soil conditions, climate, and water source [14,15]. These factors appear to produce distinct chemical compositions in the roots of American ginseng and, correspondingly, diversity in their medicinal efficacy [16]. The state of Tennessee was one of the first states where wild American ginseng was discovered and exported, and it remains the third leading state for producing wild American ginseng [17]. Despite the long history and production of wild American ginseng in Tennessee, there were limited reports on the chemical composition of authentic wild Tennessee ginseng. Tennessee’s location in the southeast portion of the Appalachian Mountain range has a unique natural climate, vegetation, and soil structure, which drives a distinct composition in the ginseng grown there. Therefore, the chemical composition of authentic wild Tennessee ginseng is worthy of further research.

Non-targeted metabolomics is one of the most powerful tools to understand the chemical composition of sample objects, as the measurement of the metabolome is the endpoint of the “omics cascade”, following genomics, transcriptomics, and proteomics [18]. Ultrahigh-performance liquid chromatography-high resolution accurate mass multistage mass spectrometry (UHPLC-HRAM-MS^n^) has become one of the most widely used techniques for its unparalleled resolving power in metabolite separation and identification [19,20]. The metabolites in the ginseng extracts show a wide diversity of molecular weight, polarity, and contents and the ion species generated in electrospray ionization (ESI) further complicate the mass spectrum [21]. Hence, the metabolomic dataset from UHPLC-HRAM-MS^n^ can be very challenging to interpret. As more than ten thousand ion features could be extracted from the raw dataset, a new approach for metabolomic analysis is needed to further process the data. In this study, we developed an in-house ginsenoside library-guided ion feature extraction procedure to facilitate the analysis and interpretation of complex metabolomic data from the UHPLC-HRAM-MS^n^ experiment. The existing online databases, such as FooDB (https://www.foodb.ca, accessed on 29 November 2022), PubChem (https://pubchem.ncbi.nlm.nih.gov/, accessed on 29 November 2022), and METLIN (https://metlin.scripps.edu/, accessed on 29 November 2022), are large databases of millions of compounds designed for various purposes. It is laborious and very challenging to narrow down the candidate compounds from a large pool of returned results for ginsenosides. A metabolite library specifically for ginseng with comprehensive information related to the chemical identity and mass spectrometric signals (e.g., isotopic mass, accurate mass for different adduct ions) is desirable for ginsenoside analysis. This study aimed to investigate the chemical profiles of wild American ginseng compared to its cultivated counterpart. All the ion features extracted were analyzed using a dataset-splitting strategy for dividing the ion features into ginsenoside and non-ginsenoside relevant matrices for further data processing. Highly discriminant ion features for metabolites were filtered out based on the statistical significance and with additional data visualization methods (i.e., heatmap and volcano plots). Both ginsenosides and non-ginsenoside metabolites were identified as potential markers for distinguishing wild and cultivated American ginseng. This study reports major chemical compositions in wild American ginseng from Tennessee, USA, which may shine a light on future research and compare the pharmacological activities of wild and cultivated American ginseng.

## 2. Results and Discussion

### 2.1. Optimization of LC-MS Conditions

**Figure 1:**
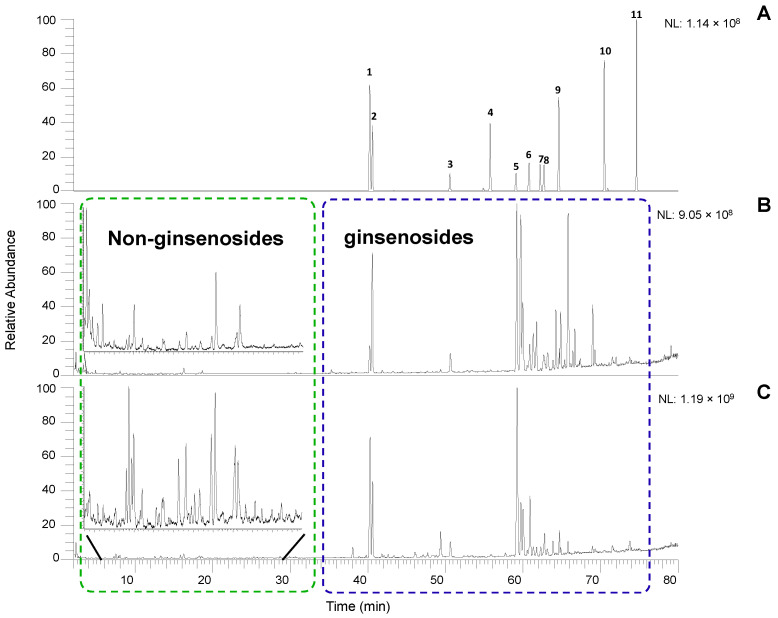
Extracted ion chromatograms of ginsenoside standard (**A**), and total ion chromatograms of wild (**B**) and cultivated (**C**) in negative ion mode (ESI^−^). The peak number labels in Figure 1A refer to the ginsenosides in Table S1. The extraction range of Figure 1A was selected as ± 0.05 Da of theoretical *m*/*z* shown in Table S1.

Based on the pre-optimized conditions of the ginsenoside reference standard mix, the mobile phase of acetonitrile/H_2_O with 0.1% formic acid with gradient elution was selected to achieve a baseline and effective separation. The quality control (QC) samples were inserted into the sequence every ten samples to guarantee the robustness of the analytical method. The peak areas of eleven known ginsenosides were monitored (Figure 1A), and their retention time reproducibility was consistent (less than 0.1 min) during the analysis. The metabolites in ginseng extracts showed good peak shapes and reproducibility in both negative and positive modes. The negative ionization mode performed better than the positive ionization mode regarding the quantity and sensitivity of the compounds of interest. However, it was still valuable to run the positive mode because some compounds showed better sensitivity than the negative mode. It also increased the confidence for the compound identification with MS data when both modes were available. The chromatographic results showed visible differences between wild and cultivated ginseng, which suggested that the metabolite profiles of the two herbal materials were distinct (Figure 1B,C).

### 2.2. XC-MS Data Preprocessing

XCMS Online is one of the most popular metabolomics data processing platforms, which integrates comprehensive statistical and visualization tools to address the challenges of converting raw high-resolution mass spectrometry datasets into interpretable results [22]. The functions incorporate raw data upload, peak detection, retention-time correction, profile alignment, comprehensive statistical data evaluation, and visualization to putative metabolite identification into a few steps, enabling high-throughput data processing for users with or without a background in the field of biostatistics. However, the peak tables obtained from this process are often overwhelmingly complicated for most researchers, requiring additional labor-intensive tasks involving significant user input. For example, a ‘feature’ refers to an ion with a unique *m*/*z* and retention time in the XCMS data processing, and the significant features are determined based on the feature’s intensity between groups with defined statistical significance (*p*-value) and magnitude of change (fold change value) criteria. With our data, 5410 metabolite features were significant (i.e., fold change ≥ 1.5 and *p*-value ≤ 0.05) to distinguish the wild and cultivated ginseng samples and output into an Excel spreadsheet and 1512 highly significant features (i.e., *p*-value ≤ 0.01) were included in the cloud plot (Figure S2). Although XCMS has effectively reduced the complexity of analyzing the UHPLC-HRMS data matrix with millions of variables into over 5000 metabolite features, further data processing is necessary to simplify identification. Therefore, we developed an in-house metabolite library for ginsenosides and related triterpene saponins to address the need for metabolite analysis in ginseng samples, especially to filter out the metabolites of interest and identify them.

### 2.3. Metabolite Feature Extraction

The identification of metabolites follows XCMS isolation of ion features in a general untargeted metabolomic workflow. In this study, several online metabolite databases were developed, which have tremendous value for compound identifications in metabolomic research. For example, XCMS provides a putative identification for each ion feature based on the METLIN database [23]. The Human Metabolome Database (HMDB) integrates detailed information about the small molecule metabolites from the human body, drug metabolites, common toxins and environmental pollutants, and food components and food additives [24]. However, these databases were designed to serve a broad spectrum of research applications in metabolomics, with a more significant emphasis on clinical and biological chemistry. The search results may lose their specificity when the study aims at a specific group of compounds (e.g., ginsenosides). For example, *m*/*z* 793.4376 was observed at 64.30 min in our ginseng LC/MS data. By searching HMDB for the possible metabolite contributing to this signal (LC/MS search settings: negative mode, unknown adduct type, and ±10 ppm tolerance), the output result includes 115 metabolites with a list of semisynthetic drugs (e.g., metildigoxin), lipids, peptides, and triterpene sapogenins. Therefore, a metabolite library specific for ginsenosides and triterpene sapogenins will greatly enhance the efficiency and effectiveness of the ginseng sample data processing by eliminating the less relevant metabolite information from the search result.

**Figure 2:**
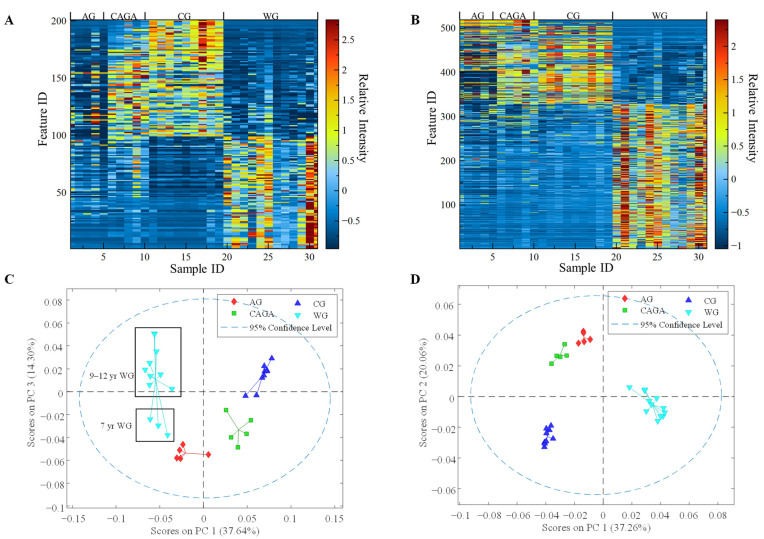
Heatmap visualization of the differential ginsenoside features (**A**) and non-ginsenoside features (**B**) in response to ginseng growing under cultivated and wild conditions, and their respective PCA score plots (**C**,**D**). The colors from blue to red in the heatmap indicate the increasing expression of ion features. The abbreviation of WG, CG, AG, and CGAG represents wild ginseng from Tennessee, cultivated ginseng from Hsu’s Ginseng Enterprise Inc., cultivated ginseng from American Herbal Pharmacopeia (AHP), and cultivated ginseng from Canadian farms, respectively.

The in-house library in this study was constructed by a literature and database survey for the ginsenosides and other triterpene sapogenins (See Table S2). In total, 468 compounds were included with their chemical name, formula, monoisotopic mass, CAS number, IUPAC name, HMDB ID, FoodB ID, and reference source. In addition, the *m*/*z* values for the common adduct ions and multiply charged ions under both positive and negative modes were calculated and included. It is known that the phytochemical profiles of American ginseng are complex, and ginsenosides are accepted as the principal and major effective components. The 5410 ion features from XCMS were searched against the common adduct ions in the in-house library with an error tolerance of ±5 mDa, and 201 ginsenoside ion matches were observed and extracted into a separate Excel spreadsheet. The variations in the 201 ginsenoside ion features with 113 unique retention times (∆t ≤ 0.2 min) among the selected ginseng species were intuitively represented by a heatmap (Figure 2A). It is worth noting that the matrix in Figure 2A was processed by autoscale, which uses mean-centering followed by a division of each variable by the standard deviation of the same variable among samples [25]. Autoscale reduces the influence of absolute ion intensity on the comparison of variables and emphasizes the signal-to-noise ratio. As shown in the heatmap (Figure 2A), the row represents the distinctive ginsenoside ion features, and each column is on behalf of different ginseng samples. The color intensity from blue to red reflects the relative intensity of each ion feature after autoscaling. A principal component analysis (PCA) score plot based on the 201 ginsenoside-related features is shown in Figure 2C. The sample clusters of the wild and cultivated ginseng groups are separated on the first principal component (PC1) coordinate direction, which accounted for 37.64% of the variance in the data. While the cultivated ginseng species could be clearly distinguished from wild ginseng, the profiles of three cultivated ginseng, including CG, AG, and CAGA, were also well-discriminated. Despite this, contrasted to the AG group, relative quantities of ginsenosides were more comparable for CG and CAGA species, which suggested that the diversity and amount of ginsenosides should be more similar. This research used a mix of commercially available cultivated ginseng samples from different sources and wild American ginseng with various ages (i.e., 7–12 years), which are a good sample representation to study the variations of metabolite compositions of American ginseng.

In addition to ginsenosides, other metabolites such as amino acids, phenolic acids, and organic acids are also important in discriminating between the metabolite profiles of ginseng grown in different regions [26]. In order to further reveal metabolite profile differences, the non-ginsenoside metabolite matrix was also studied to compare cultivated and wild American ginsengs. The non-ginsenoside features were obtained by subtracting the ginsenoside features from the extracted features mentioned above. The non-ginsenoside features with a *p*-value cutoff of 0.05 and a change of at least 2-folds were selected for further heatmap analysis to select the highly significant features. As shown in Figure 2B, the 519 non-ginsenoside features with 210 unique retention times showed the distinctive variables responsible for the compounds between the wild and cultivated ginseng samples. The PCA score plot (Figure 2D) was constructed based on the 519 non-ginsenoside features. The differentiation is consistent with the results from ginsenoside features, with a more apparent separation between cultivated and wild ginseng groups.

**Figure 3:**
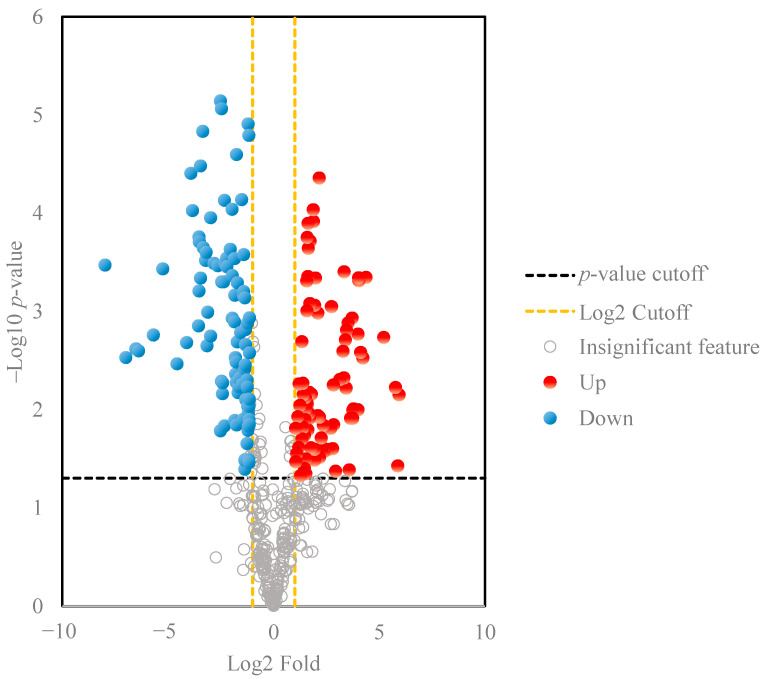
Volcano plots results of ginsenoside features in the ginseng samples. The order of the metabolites on the y-axis is determined by the statistical significance *p*-value of the ratio fold-change.

A volcano plot was also used for data visualization to discover independent changes in ginsenoside levels and discriminate between wild ginseng and cultivated ginseng samples. The 201 important ginsenoside ion features with selective fold change threshold (>2) and *t*-tests threshold (*p* < 0.05) as cutoff values for volcano plots to assist in removing statistically less significant features. Ginsenosides that have a relatively low fold-change between these two sample sets are close to the central vertical y-axis; metabolites that have a higher fold-change are found in the upper-right or upper-left. The results indicated that compared with the cultivated ginseng group, the content of the 99 selective ginsenoside ion features (red circles) in the wild ginseng group was in greater abundance than the cultivated ginseng, and that of 102 features (blue features) was in greater abundance in the cultivated ginseng compared to the wild ginseng (Figure 3). It is worth noting that multiple ion features may correlate with the same ginsenoside metabolites due to the co-existence of the common adduct ions and multiply charged ions in the ESI-MS spectrum. So the number of significant ginsenoside metabolites is much smaller than 201. For example, *m*/*z* 561.2908, 584.2938, 1077.5833, 1123.5900, and 1191.5775 at 62.7 min were observed in the ginsenoside Rc full scan spectrum, which corresponded to [M-2H]^2−^, [M + Formic Acid-2H]^2−^, [M-H]^−^, [M+ Formic Acid-H]^−^, and [M+ Trifluoroacetic Acid -H]^−^. Though all these ions were formed from ginsenoside Rc molecules during the ESI process, they were considered unique features from the XCMS data preprocessing. With the ginsenoside library provided, these ion features can be easily combined to reduce the workload of ginsenoside identification in the next step.

### 2.4. Classification and Distribution of Ginsenosides

The typical structure of a ginsenoside involves a triterpene sapogenin and one or multiple sugar units. As known, protopanaxadiol (PPD), protopanaxatriol (PPT), octillol (OT), and oleanolic acid (OA) types are found to be the most common sapogenins for ginsenosides [27]. Until now, hundreds of ginsenosides have been isolated and unambiguously characterized from *Panax* species using MS technology, [26,28] which makes it possible to identify the ginsenosides based on the mass fragmentation pathways from the samples. The characteristic fragment ions at *m*/*z* 459, 475, 491, and 455 were attributed to the PPD-, PPT-, OT- and OA-type aglycones, respectively. Based on the above rule, the ion mapping function of HRMS was performed to extract all the parent ions yielding products with 459, 475, 491, and 455 amu. As a result, 56 ginsenosides characterized as PPD (17), PPT (28), OT (6), and OA (5) categories were extracted and identified (Table S3). The result agrees with the current knowledge that PPD and PPT-type saponins account for the dominant ginsenosides in American ginseng. [29]

**Figure 4:**
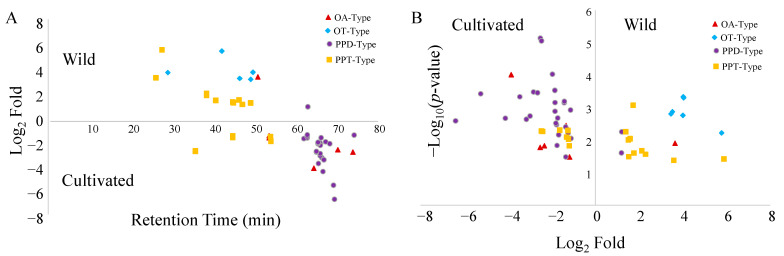
Distribution trends of ginsenoside types based on retention times (**A**) and growth environments (**B**).

To obtain a more intuitive presentation, the distribution trends of different ginsenoside types between cultivated and wild ginseng samples were further characterized based on retention times and quantitative variations by volcano plots. As indicated in Figure 4B, the number of PPD-, PPT-, OT-, and OA-type ginsenosides, which are more abundant in wild ginseng, is 2, 10, 6, and 1, respectively. The corresponding number of ginsenosides more favorable in cultivated ginseng is 26, 7, 0, and 4, respectively. In other words, the number of PPD- and OA-types showed an advantage in cultivated ginsengs; however, the dominant OT-types were specifically exiting in wild ginsengs. Interestingly, the abundant ginsenosides in cultivated ginsengs were subsequently eluted after 50 min; conversely, the corresponding ginsenosides in wild ginsengs primarily appeared before 50 min (Figure 4A). It could be speculated that wild American ginsengs are rich in polar ginsenosides, yet those are less polar in cultivated ginseng. Additionally, if not considering the difference of glycosyl substituents, the polarities of PPT-type ginsenosides tend to be higher than those of PPD-type due to the existence of one more hydroxyl group in PPT-type aglycon than in PPD-type aglycon. This point is consistent with our conclusion that wild-type ginsengs are more abundant in PPT-type ginsenosides and less in PPD-type ginsenosides.

The synthesis of ginsenosides is related to the growth environment and key enzyme genes. For instance, photosynthetically active radiation (PAR) responded positively to PPT-type ginsenosides, which showed a direct correlation with light [30,31]. This partially clarified the reason why, compared with cultivated ginseng, those growing in the wild showed more abundant PPT-type ginsenosides (Figure 4). It is speculated that the growth environments between cultivated and wild ginseng are different (e.g., shading, soil water potential, relative humidity, and rain), and our results support that the composition of individual ginsenosides is different depending on the various growth environment [32]. It is a circumstantial basis that the variation of ginsenoside profiles was observed within cultivated ginseng obtained from different sources (e.g., CG, AG, and CGAG in Figure 2). Interestingly, it was reported that the ratio of PPD-type ginsenosides (Rb1, Rb2, Rc, and Rd) to PPT-type ginsenosides (Rg1, Re, and Rh1) changed during different growth stages [30]. Generally, cultivated ginseng roots are harvested after a 4–6-year cultivation period [33]. However, wild ginseng is typically harvested for between 7–12 years [34]. As a plant on the list of the Convention on International Trade in Endangered Species of Wild Fauna and Flora (CITES), the wild ginseng roots cannot be legally exported if they were harvested at less than 5 years old. Some states, including Illinois, Vermont, and the Menominee Indian Tribe of Wisconsin, require wild ginseng plants to have 4 leaves and to be 10 years old [35]. The harvest age requirements for cultivated and wild American ginseng are different. Therefore, selecting samples at their normal harvest ages, though different, should be practically more representative in their regular use. To make the results reliable, we selected the cultivated ginseng from different merchants and the wild ginseng harvested for a range of years. Interestingly, we still found the secondary metabolite profile discrimination between wild and cultivated ginseng from the selected sample set. The results strongly suggested that the growth environment makes a statistically significant difference in the metabolites profile. Therefore, all these increased the complication of distinguishing the metabolites profile of ginseng growing under different environments and harvesting at various stages. In fact, we also found the ginseng samples aged from 7 to 12 years may be differentiated based on their detected metabolite profile (Figure S3). As a result, more research with a larger sample size is needed to clarify the complicated profile variation further. It has been expected that the metabolite profile of ginseng performed age discrimination [33,36]. In addition, previous research has shown that soils in the farmlands of cultivated ginseng have significantly different biodiversity compared to wild soil samples [37]. Ginseng also interacts with nearby plants, [38] fungi, [39] and microbes [40], indicating that the more diverse biomes surrounding the wild samples may play a role in the metabolomic differences that we see in this study.

### 2.5. Identification and Qualification of Marker Ginsenosides

The most discriminative ginsenoside ion features in the volcano plots (Figure 3) were extracted and identified with the in-house ginsenoside library, reference standards, and knowledge of MS fragmentation behavior [27,33,41,42,43]. The MS fragmentation mechanism of ginsenoside is elucidated as follows: the deprotonated ions [M−H]^−^ and customary adduct ions [M+HCOO]^−^ were often monitored under ESI^-^ mode, which would allow us to obtain the monoisotopic mass and formula of the compounds with the accurate HRMS data. The characteristic sugar units can be characterized by the loss of 162 Da (-Glc), 132 Da (-Xyl/Ara), 146 Da (-Rha), or 176 Da (-GlurA). The common substituents such as acetyl (Ace), butenoyl (But), or malonyl (Mal) units attached at the hydroxy groups would be characterized by the detaching of 42 Da (-Ace), 68 Da (-But), and 86 Da (-Mal). Sugar chains first lose the acylation moieties and then the glycosyls. As mentioned above, the neutral losses of characteristic ions, 459 Da, 475 Da, 491 Da, and 455 Da, were assigned to PPD-, PPT-, OT-, and OA-type aglycones, respectively. Based on the strategies described above, eleven ginsenoside ion features with high statistical significance were tentatively identified (Table 1), of which five (notoginsenoside H, glucoginsenoside Rf, notoginsenoside R1, pseudoginsenoside RT2, ginsenoside Rc) were more abundant in wild type ginseng, and the other six (chicusetsusaponin IVa, malonylginsenoside Rd, pseudoginsenoside Rc1, malonylfloralginsenoside Rd6, Ginsenoside Rd, and malonylginsenoside Rb1) more favorably appeared in cultivated types. Among them, ginsenoside Rc and Rd were confirmed by reference standards, and others were characterized using the MS fragmentation behaviors and compared with our in-house library and existing literature [27,33,41,42,43,44,45].

**Figure 5:**
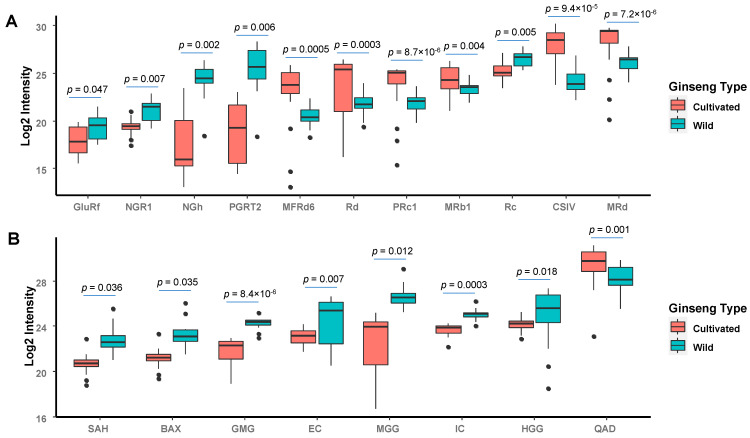
Comparison of identified marker ginsenoside (**A**) and non-ginsenoside (**B**) compounds in wild and cultivated ginseng samples. The semi-quantification was performed based on the normalized ion intensity obtained from HRMS. Black dots are outliers.

The effects of the growth environment on marker ginsenosides were also quantitatively evaluated using relative ion intensities. As shown in Figure 5A, the distinctive ginsenosides in wild-type ginsengs were a 2.3–54.5-fold higher compared to cultivated ginseng. In addition to the most common dammarane-types, OT-type saponins such as notoginsenoside H and pseudoginsenoside RT2 occupied a notable proportion with 15.9- and 54.5-folds higher than cultivated types. The OT-type ginsenosides are characteristic compounds for American ginseng that are distinguished from other species, such as Asian ginseng [41,46]. Our results further confirmed that the corresponding OT-type ginsenosides were significantly higher in the wild American ginseng. The results suggested that the characteristic OT-type ginsenosides with notable distribution could be the marker compounds for the wild type of American ginseng to distinguish from cultivated and/or other ginseng species. Conversely, the content of ginsenosides dominated exiting in cultivated type ginsengs was 2.6–14.4-folds higher compared to wild ginsengs. Among these, most of the markers were assigned to PPD-type, and only one was identified as OA-type ginsenoside. It is notable that, among the marker compounds, malony-ginsenosides are rich in cultivated ginsengs, yet higher levels of the minor ginsenosides were found in wild ginseng (Figure 5A). The finding was further confirmed by a previous report that, among American ginseng, wild samples often had more of the notoginsenosides R1 and Rw2 and less abundance of the ginsenosides Rd, Rd isomer, and 20 (S)-Rg3 than cultivated samples [47]. The possible explanation is that the malonyl-ginsenosides are the precursor ginsenosides which could be converted into other minor ginsenosides through hydrolysis, de-glycosylation, dehydration, and acetylation during growth circumstances in wild ginseng [48].

Different types of ginsenosides may perform distinctively different pharmacological activities. For example, notoginsenoside R1, abundant in wild ginseng, exhibits various biological activities such as cardiovascular protection, [49] neuroprotection, [50] anti-diabetes, [51] and bone metabolism regulation [52]. However, Ginsenoside Rd, rich in cultivated ginseng, performs significant roles in antifibrotic activity, [53] anti-inflammatory activity, [54] and anxiolytic activity [55]. Similarly, OT-type ginsenosides such as notoginsenoside H have been shown to have neurological and cardiovascular protective effects, along with anticancer, antibacterial, and anti-inflammatory effects [56]. In comparison, OA-type sapogenins have been shown to have positive effects on metabolic syndrome disorders such as diabetes and high cholesterol by improving insulin response and decreasing levels of total cholesterol in the blood, specifically triglycerides and low-density lipoproteins [57]. Since these sapogenins are asymmetrically distributed between our wild and cultivated samples, a greater understanding of the molecular mechanisms driving their biosynthesis could provide insight to produce a more pharmacologically important product. Our results will provide the chemical basis directly related to their pharmacological activities, thus resulting in the different clinical applications of wild and cultivated ginseng.

### 2.6. Identification and Quantification of Other Marker Metabolites

In addition to ginsenosides, non-ginsenoside metabolites also play important roles in distinguishing ginseng species. For example, Lin et al., reported that the content of organic acids such as palmitoleic acid and α-linolenic acid were higher in field-grown American ginseng, yet the content of methyl gallate glucoside was more abundant in wild-simulated ginseng, which would be the potential markers to differentiate wild-simulated and field-grown American ginseng [46]. Therefore, we further identified and semi-quantified the non-ginsenoside profile of investigated American ginseng samples. As a result, thirteen non-ginsenoside metabolites (e.g., sucrose, amino acid, organic acid and derivatives, and disaccharide derivatives) were identified from 0–20 min, as most of the significant non-ginsenoside metabolites were eluted within this time range (Table S4). The semi-quantification was taken based on the relative ion intensity, and important compounds with selective fold change threshold (>2) and *t*-tests threshold (*p* < 0.05) were selected (Table 2 and Figure 5B). Interestingly, quinic acid derivative was found to be 2.6-folds higher in the cultivated type of ginseng than in the wild type. However, seven marker compounds (i.e., methyl gallate-glucoside, sinapic acid hexoside, everlastoside C, isoconiferoside, and three disaccharide derivatives) exiting in wild ginsengs were 2.6–9.2-folds higher compared to those in the cultivated ginsengs (Figure 5B). Therefore, these non-ginsenoside metabolites could also be considered as major marker compounds that could discriminate wild type and cultivated America ginseng. It is known that ginsenosides are primarily responsible for the pharmacological actions of ginseng; however, non-ginsenoside metabolites are also important to the function of ginseng. For example, Jang et al., confirmed that organic acids in ginseng constitute a significant factor affecting ginsenoside conversion [58]. As well as indirect effects, non-ginsenoside metabolites also perform a direct role in ginseng activity; e.g., Dong et al. reported that malic acid and quinic acid were suggested to be pharmacodynamic markers of American ginseng against heart failure [59]. Thus, in addition to ginsenosides, the knowledge of marker non-ginsenoside metabolites is necessary to understand the chemical variety and further reveal the basis of distinctive pharmacological activities between wild and cultivated ginseng.

## 3. Materials and Methods

### 3.1. Chemicals and Reagents

Formic acid and high-performance liquid chromatography (HPLC)-grade methanol, acetonitrile, and water were purchased from Fisher Scientific (Waltham, MA, USA). The ginsenoside reference standards, including Rb1, Rb2, Rb3, Rc, Rd, Rg1, Rg2, Rg3, Re, A1, and Rh2, were obtained from Sigma-Aldrich (MO, USA) and prepared to the mixture of 20 µg/mL using methanol–water (7:3, *v*/*v*).

### 3.2. Sample Collection

The 12 wild ginseng roots (7, 9, 10, and 12 years, *n* = 3) were collected from the Appalachian regions of Eastern Tennessee, and their ages were estimated via counting root scars. Nine fresh 5-year-old cultivated American ginseng roots were purchased from Hsu’s Ginseng Enterprise Inc. (Wausau, WI, USA) and freeze-dried in the lab before extraction. Five dried cultivated ginseng roots were obtained from American Herbal Pharmacopeia (AHP, USA), and five were purchased from Canadian ginseng farms online. The abbreviation of WG, CG, AG, and CGAG represents wild ginseng from Tennessee, cultivated ginseng from Hsu’s Ginseng Enterprise Inc., cultivated ginseng from American Herbal Pharmacopeia (AHP), and cultivated ginseng from Canadian farms, respectively.

### 3.3. Sample Processing and Extraction

The fresh samples were set out for eight days in a Thermo Fisher Scientific MaxQ 4000 incubator (Waltham, MA, USA) set to 30 °C to dry the cultivated roots. After drying, roots were stored in the −80 °C freezer until ready for homogenization. For both the cultivated and wild roots, samples were initially submerged in liquid nitrogen and homogenized by a blade grinder until powdered. After homogenization, samples were portioned out and lyophilized by Labconco Freezone 2.5 plus (Labconco Corporation, Kansas City, MO, USA) for 24 h. Samples were then measured and extracted by a solvent with 70% UHPLC grade methanol and 30% UHPLC grade water (*v*/*v*). The extraction was carried out with 4× the extraction solvent in mL per gram of lyophilized sample in a 5-mL Eppendorf tube, with the mixture tapped to a Thermo Fisher Scientific digital mini rotator (Waltham, MA, USA) set to 500 rpm for one hour. Afterward, the sample was centrifuged at 20,000× *g* for 10 min in a refrigerated centrifuge under 4 °C, followed by syringe filtration through a 0.22 µm filter. Lastly, samples were evaporated under nitrogen and resuspended in equivalent volumes of extraction solvent. Processed samples were stored in a −80 °C freezer and were diluted 10-fold using extraction solvent before the UHPLC-HRMS analysis.

### 3.4. UHPLC-HRMS Analyses

The UHPLC-HRMS consisted of a Vanquish UHPLC and Q Exactive mass spectrometer (Thermo Fisher, Waltham, MA, USA) with an electrospray ionization source. A full mass range was set at *m*/*z* 120–1800 with a resolution of 70,000. A data-dependent MS^2^ acquisition method was constructed for the top five intense ions. AGC values were set to 3 × 10^6^ for MS and 1 × 10^5^ for MS/MS. The mass injection time was set as 85 min. The ESI source was used under both positive and negative modes with a spray voltage of 4000 V, respectively. Sheath gas, aux gas, and sweep gas were set at 40, 10, and 5 (arbitrary unit), respectively. The ion transfer tube temperature was set at 300 °C.

The separation was carried out on a Thermo Hypersil Gold AQ RP- C_18_ UHPLC column (200 mm × 2.1 mm i.d., 1.9 µm) (ThermoFisher Scientific, Waltham, MA, USA) with an UltraShield pre-column filter (Analytical Scientific Instruments, Richmond, CA, USA) at a flow rate of 0.3 mL/min. The mobile phase consisted of a combination of A (0.1% formic acid in water, *v*/*v*) and B (0.1% formic acid in acetonitrile, *v*/*v*). The column was pre-equilibrated with 2% organic phase for 10 min. Then, the linear gradient was from 2% to 15% B (*v*/*v*) at 20 min, to 35% B at 60 min, to 55% B at 70 min, to 95% B at 80 min, and maintained 95% B till 85 min. The UV wavelength was set at 280 nm, 203 nm, and 350 nm to record the peaks. The injection value was set as 1 µL.

### 3.5. Data Pretreatment

Raw files from UHPLC-HRMS were converted into the mzXML format using Proteowizard 3.0.20210 (http://proteowizard.sourceforge.net/, accessed on 29 November 2022), and then, XCMS Online (https://xcmsonline.scripps.edu/, accessed on 29 November 2022) was selected for advanced data processing [67]. Pairwise analysis was selected, and the data were grouped into two categories: wild American ginseng and cultivated American ginseng. The steps included ion feature extraction, peak picking, and alignment with the following settings: a centWave feature detection method with a ppm value of 5, and snthr value of 4, peak width value of 5.20, mzdiff value of 0.01, prefilter peak value set to 3, and prefilter intensity set to 100. Additional settings included retention time correction utilizing an obiwarp method with a profStep value of 1, grouping using a density method with a bw, mzwid, minfrac, and minsamp values set to 5, 0.025, 0.5, and 1, respectively. Statistics included a Welch *t*-test, with a *p*-value threshold for highly significant features: 0.01, fold change threshold of highly significant features: 1.5, and *p*-value threshold for significant features: 0.05. The ion feature list was downloaded in an Excel Spreadsheet for further analysis.

### 3.6. In-House Library Construction and Application

The in-house library was constructed by collecting ginsenosides and other steroid compounds in the existing literature and database (i.e., FoodB). In total, 468 compounds were included in our in-house library, and the complete list of these compounds is provided in the supplementary materials (Table S2). For each record, the chemical name, formula, CAS number (if available), FoodB ID (if available), HMDB ID (if available), source of information, accurate mass, and the *m*/*z* of possible adduct ions and multiply charged ions in both positive and negative modes, are provided.

The *m*/*z* in the ion feature list from the XCMS analysis was compared with the *m*/*z* in the in-house database to screen the possible ginsenosides, and if the ∆*m*/*z* is within the ±5 mDa, the ion feature is flagged and considered as a potential ginsenoside. This process was conducted using MATLAB R2021a (MathWorks Inc., Natick, MA, USA). The extracted ginsenoside ion features were exported into an Excel spreadsheet and verified manually. The remaining ion features from the XCMS analysis were used as non-ginsenoside features. The chemometric analysis was conducted using MATLAB R2021a (MathWorks Inc., Natick, MA, USA) with the PLS toolbox (Eigenvector Research, Inc., Manson, WA, USA).

### 3.7. Statistical Analysis

The ion features with selective fold change threshold (>2) and t-tests threshold (*p* < 0.05) were selected to generate the volcano plots using Microsoft Excel (Microsoft Corporation, Redmond, WA, USA). Both fold changes and *p*-values were log-transformed, and the further its position away from the (0, 0), the more significant the feature is. Statistical analysis was carried out using Student’s *t*-test function available in the Microsoft Excel software (Microsoft Corporation, Redmond, WA, USA). The *p* values less than 0.05 were considered statistically significant.

## 4. Conclusions

In summary, our study elaborated on the secondary metabolite profile differences between wild and cultivated America ginseng using an untargeted UPLC-HRMS-based metabolomic approach. Ginseng samples from different sources were successfully discriminated based on their ginsenoside and non-ginsenoside metabolite profiles. Data were further processed by using the in-house ginsenoside library, and 56 marker ginsenosides were discovered to be responsible for the distinction. Among them, PPD-type (e.g., malonyl-ginsenosides) are more abundant in cultivated type ginsengs, yet higher levels of the OT-type ginsenosides (e.g., notoginsenoside R1, pseudoginsenoside RT2, and ginsenoside Rc) were found in wild ginseng. Additionally, the non-ginsenoside metabolites, such as organic acid derivatives, were also responsible for the discrimination. Future work will include evaluating how the different ages and environmental conditions result in the metabolomic differences in American ginseng. Our results suggested that using the identified characteristic components as chemical markers to identify cultivated and wild America ginseng is effective and viable, and the strategy would be beneficial for the quality evaluation of America ginseng.

## Figures and Tables

**Table 1:** Discriminative ginsenosides tentatively identified between cultivated and wild ginseng.

Peaks	t_R_ (min)	ESI (− Measured (*m*/*z*)	Adduct	Neutral Formula	Error (ppm)	Major MS^2^ Ions (100%)	Tentative Identification	Aglycone Type	Abbreviation	References
1	28.6	993.5275	−H, +HCOO	C_47_H_80_O_19_	1.12	[993]: 947(100), 815(58), 797(8), 653(94), 491(6), 191(6), 179(14), 161(69)	notoginsenoside H or isomer	OT	NGH	[27]
2	34.6	1007.545	−H, +HCOO	C_48_H_82_O_19_	2.10	[1007]: 961(100), 799(28), 781(6), 637(34), 475(11), 179(15), 161(30)	glucoginsenoside Rf or isomer	PPT	GluRf	[42]
3	38.5	977.5331	−H, +HCOO	C_47_H_80_O_18_	1.28	[977]: 931(94), 799(38), 637 (100), 161(32)	notoginsenoside R1 or isomer	PPT	NGR1	[33]
4	49.4	831.4748	−H, +HCOO	C_41_H_70_O_14_	−1.85	[831]: 785(88), 653(73), 191(7), 161(100)	pseudoginsenoside RT2 or isomer	OT	PGRT2	[42]
5	62.8	1123.591	−H, +HCOO	C_53_H_90_O_22_	−1.14	[1123]: 1078(100), 945(16), 916(9), 783(14), 191(9)	ginsenoside Rc ^#^	PPD	Rc	[43]
6	64.3	793.4376	−H	C_42_H_66_O_14_	−0.53	[793]: 793(100), 631(20), 569(7), 455(3)	chicusetsusaponin IVa or isomer	OA	CSIV	[43]
7	64.9	1031.542	−H	C_51_H_84_O_21_	−0.25	[1031]: 945(100), 927(17), 783(22), 765(14), 663(5), 621(15), 459(4)	malonylginsenoside Rd or isomer	PPD	MRd	[43]
8	64.9	987.5515	−H	C_50_H_84_O_19_	−0.99	[987]: 945(100), 927(13), 783(25), 765(15), 663(6), 621(21), 459(5), 179(10), 161(32)	pseudoginsenoside Rc1 or isomer	PPD	PRc1	[42]
9	65.4	1117.545	−H	C_54_H_86_O_24_	0.84	[1117]: 987(35), 945(100), 927(78), 783(23), 765(30), 621(25)	malonylfloralginsenoside Rd6 or isomer	PPD	MFRd6	[42]
10	65.8	991.5477	−H, +HCOO	C_48_H_82_O_18_	−0.56	[945]: 945(100), 783(20), 621(11), 459 (3)	ginsenoside Rd ^#^	PPD	Rd	[43]
11	62.6	1193.595	−H	C_57_H_94_O_26_	0.62	[1193]: 1107(100), 1089(38), 945(25), 927(6), 783(15), 179(39)	malonylginsenoside Rb1 or isomer	PPD	MRb1	[45]

^#^, confirmed by reference standards.

**Table 2:** Discriminative non-ginsenosides tentatively identified between cultivated and wild ginseng.

Peaks	t_R_ (min)	ESI (−) Measured (*m*/*z*)	Adduct	Neutral Formula	Error (ppm)	Major MS^2^ Ions (100%)	Tentative Identification	Abbreviation	References
1	1.8	533.1718	−H	C_19_H_34_O_17_	−0.83	[533]: 191(100)	quinic acid derivative	QAD	[60]
2	7.5	345.0816	−H	C_14_H_18_O_10_	0.12	[345]: 345(90), 330(100), 183(55)	methyl gallate-glucoside	MGG	[46]
3	13.4	471.2087	−H, +HCOO	C_18_H_34_O_11_	0.91	[471]: 425(100), 293(91), 161(26)	hexyl 6-O-glucopyranosyl-glucopyranoside	HGG	[61]
4	14.0	425.1672	−H	C_17_H_30_O_12_	1.86	[425]:379(20), 191(8), 179(48), 161(12), 101(31), 89(100)	glucopyranosyl-methylbutanoyl-glucopyranoside	GMG	[62]
5	14.3	385.1144	−H	C_17_H_22_O_10_	0.84	[385]: 223(100), 208(18), 179(47), 164(36)	sinapic acid hexoside	SAH	[63]
6	14.8	447.1513	−H, +HCOO	C_18_H_26_O_10_	0.55	[447]: 401(13), 269(100), 161(42)	benzyl alcohol xylopyranosyl-(1–6)-glucopyranoside	BAX	[64]
7	15.9	381.1767	−H	C_16_H_30_O_10_	0.21	[381]:249(100), 161(34), 101(39), 89(13)	everlastoside C	EC	[65]
8	18.2	503.1774	−H	C_22_H_32_O13	0.77	[503]: 503(100), 341(18), 179(10), 161(43), 101(46),89(41)	isoconiferoside	IC	[66]

## Data Availability

The data presented in this study are available on request from the corresponding author.

## Supplementary Materials

The following supporting information can be downloaded at: https://www.mdpi.com/article/10.3390/molecules28010009/s1, Table S1: Characterization of ginsenoside reference standards by UHPLC-HRMS; Table S2: In-house ginsenoside library; Table S3 Summary of ginsenoside distribution; Table S4 Discriminative non-ginsenosides tentatively identified between cultivated and wild ginseng; Figure S1: The chemical structures of the common ginsenosides in American ginseng; Figure S2: Cloud plot from XCMS with highly significant metabolite features (i.e., fold change ≥ 1.5 and *p*-value ≤ 0.01) labeled as circles. The green and red colors represent the up-regulated and down-regulated metabolites in the wild and cultivated ginseng samples, respectively, and statistical significance (*p*-value) is represented by the bubble’s color intensity. The size of the bubble denotes feature intensity; Figure S3: Partial least squares-discriminant analysis (PLS-DA) of metabolites profiles of wild ginseng (WG) harvested at 7, 9, 10, and 12 years old.
