# Supplementary material for: Comparison of Phytochemical Profiles of Wild and Cultivated American Ginseng Using Metabolomics by Ultra-High Performance Liquid Chromatography-High-Resolution Mass Spectrometry
Source: Molecules. 2022 Dec 20;28(1):9. doi: 10.3390/molecules28010009 (PMC9821851; doi:10.3390/molecules28010009)
Supplement: Supplementary file 1 [file molecules-28-00009-s001.zip › Supplementary figures.pdf]

Supplementary figures

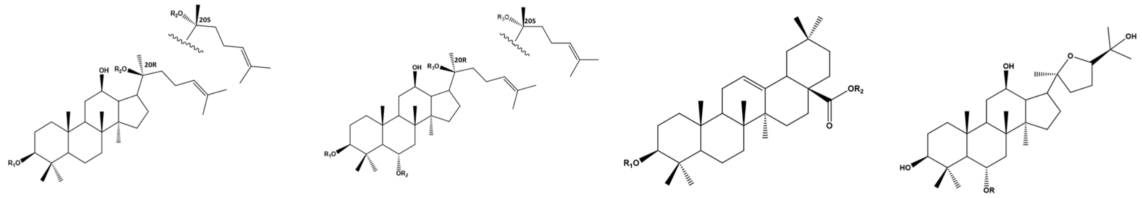

Protopanaxadiol-Type (PPD)

Ginsenoside Rb1 R_1= 2Glc, R_2=2Glc
Ginsenoside Rb2 R_1= 2Glc, R_2=Glc-Ara(p)
Ginsenoside Rb3 R_1= 2Glc, R_2=Glc-Xyl
Ginsenoside Rc R_1= 2Glc, R_2=Glc-Ara(p)
Ginsenoside Rd R_1= 2Glc, R_2=Glc
Ginsenoside Rg3 R_1= 2Glc, R_2=H
Ginsenoside Rh2 R_1= Glc, R_2=H
Malonylginsenoside Rd R_1= 2Glc-Mal, R_2=Glc
Pseudoginsenoside Rc1 R_1= 2Glc-Ace, R_2=Glc
Malnoylfloralginsenosides Rd6 R_1= 2Glc-Mal, R_2=Glc-Mal
Malonylginsenoside Rb1 R_1= 2Glc-Mal, R_2=Glc

Protopanaxatriol-Type (PPT)

Ginsenoside Rg1 R_1=H, R_2=R_3=Glc
Ginsenoside Re R_1=H, R_2=2Glc, R_3=Glc
Ginsenoside Rg2 R_1=R_3=H, R_2= 2Glc
Notoginsenoside R1 R_1= Glc, R_2=Glc-Xyl, R_3=Glc
Glucoginsenoside Rf R_1= H, R_2=2Glc, R_3=Glc

Oleanolic Acid-Type (OA)

Chicusetsusaponin IVa R_1=GlurA, R_2=Glc
Ginsenoside Ro R_1=GlurA-Xyl, R_2=Glc

Octillol-Type (OT)

Pseudoginsenoside-RT2 R=Glc-Xyl
Pseudoginsenoside F11 R-Glc

Figure S1 The chemical structures of the common ginsenosides in American ginseng

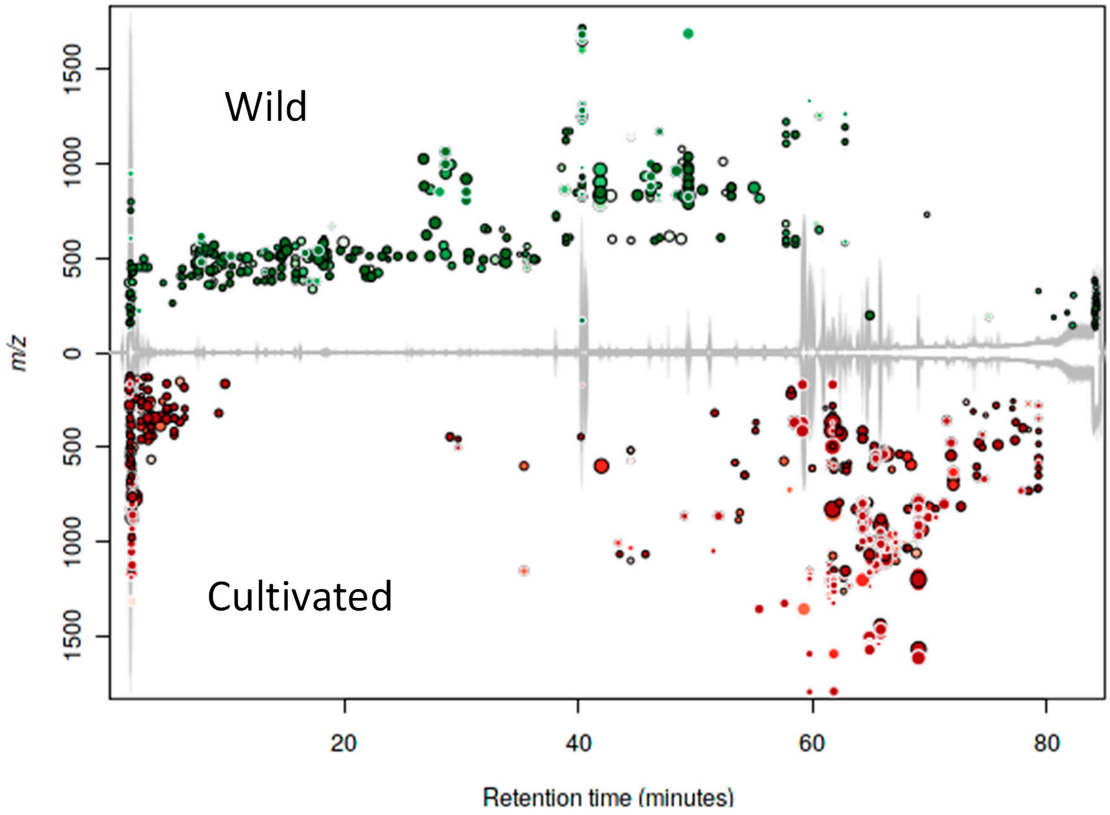

Figure S2. Cloud plot from XCMS with highly significant metabolite features (i.e., fold change ≥ 1.5 and p-value ≤ 0.01) labeled as circles. The green and red colors represent the up-regulated and down-regulated metabolites in the wild and cultivated ginseng samples, respectively, and statistical significance (p-value) is represented by the bubble's color intensity. The size of the bubble denotes feature intensity.

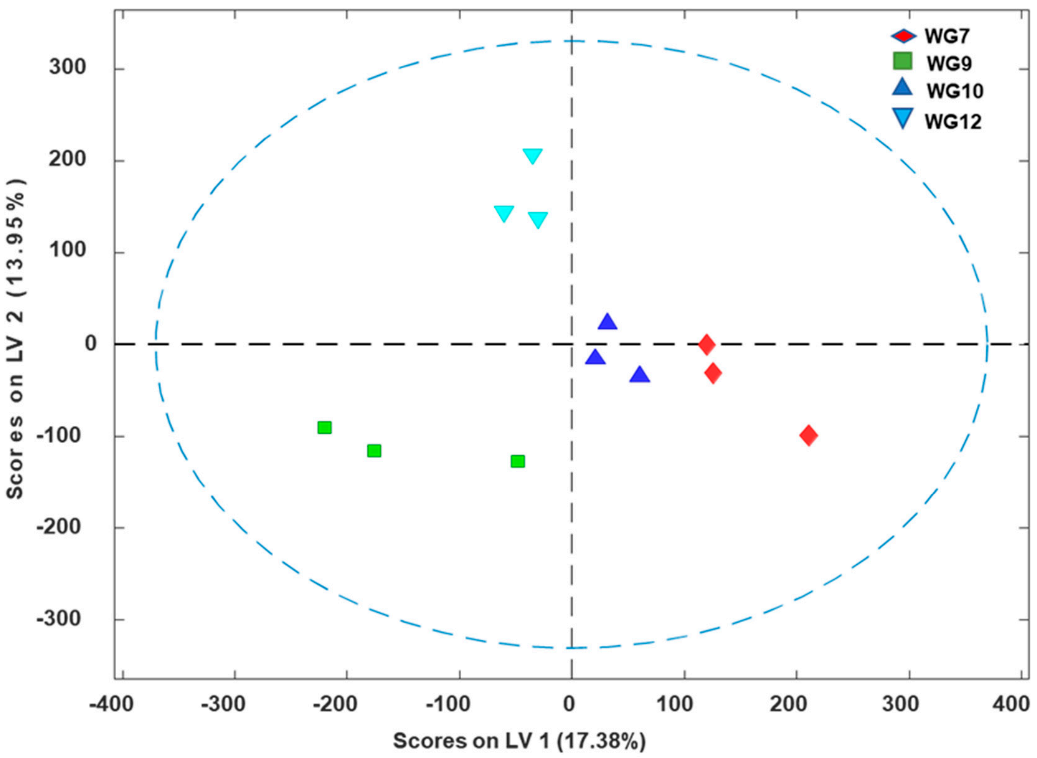

Figure S3. Partial Least Squares-Discriminant Analysis (PLS-DA) of metabolites profiles of wild ginseng (WG) harvested at 7, 9, 10, and 12 years old.
