# Supplementary material for: Comparison of Phytochemical Profiles of Wild and Cultivated American Ginseng Using Metabolomics by Ultra-High Performance Liquid Chromatography-High-Resolution Mass Spectrometry
Source: Molecules. 2022 Dec 20;28(1):9. doi: 10.3390/molecules28010009 (PMC9821851; doi:10.3390/molecules28010009)
Supplement: Supplementary file 1 [file molecules-28-00009-s001.zip › Supplementary tables-Table S1, S3 and S4.pdf]

Table S1 Characterization of Ginsenoside reference standards by UHPLC-HRMS

Peaks	Rt(min)	Ginsenosides	Formula	Error(ppm)	[M-H]$^-$	[M+FA-H]$^-$	Major MS^2 ions (100%)
1	40.2	Ginsenoside Rg1	$C_{42}H_{72}O_{14}$	-0.23	799.4847	845.4898	[845]: 637(56), 475(32), 179(15), 161(59), 89(100)
2	40.6	Ginsenoside Re	$C_{48}H_{82}O_{18}$	-1.23	945.5434	991.5476	MS2[991]: 945(100), 799(15), 783(32), 765(12), 637(52), 619(16), 475(20), 179(18), 161(42), 101(48), 89(59)
3	50.6	Pseudoginsenoside F11 (Ginsenoside A1)	$C_{42}H_{72}O_{14}$	0.15	799.485	845.4904	[845]: 799(100), 653(30), 161(34), 113(13), 101(30), 491(4)
4	55.8	Ginsenoside Rg2	$C_{42}H_{72}O_{13}$	0.27	783.4902	829.495	[829]: 783(90), 637(41), 619(11), 475(61), 205(19), 161(83), 159(12), 119(24), 113(40), 101(100), 89(44)
5	59.1	Ginsenoside Rb1	$C_{54}H_{92}O_{23}$	-0.07	1107.596	1153.601	[1153]: 1107(100), 945(24), 783(14), 179(25), 101(13), 89(12)
6	60.8	Ginsenoside Rb2	$C_{53}H_{90}O_{22}$	-1.60	1077.583	1123.59	[1123]: 1077(100), 945(24), 783(22), 191(26), 149(26), 131(12), 101(14), 89(23)
7	62.3	Ginsenoside Rb3	$C_{53}H_{90}O_{22}$	-1.71	1077.583	1123.59	[1123]: 1077(100), 945(15), 783(16), 149(34), 101(14), 89(20)
8	62.7	Ginsenoside Rc	$C_{53}H_{90}O_{22}$	-1.37	1077.584	1123.59	[1123]: 1077(100), 945(16), 783(18), 149(35), 101(16), 89(22)
9	64.6	Ginsenoside Rd	$C_{48}H_{82}O_{18}$	-2.12	945.5408	991.5471	[991]: 945(100), 783(28), 621(16), 161(20), 119(13), 101(23), 89(21), 71(14), 459(3)
10	70.6	Ginsenoside Rg3	$C_{42}H_{72}O_{13}$	-0.03	783.49	829.4948	[829]: 783(100), 621(25), 459(11), 161(74), 159(13), 143(13), 119(14), 113(48), 101(73), 89(30)
11	74.7	Ginsenoside Rh2	$C_{36}H_{62}O_{8}$	-0.78	621.4412	667.442	[667]: 621(45), 528(21), 459(49), 345(22), 215(21), 161(100), 118(19), 113(59), 101(94)

Table S3 Summary of Ginsenoside Distribution

	Wild	Cultivated	Total
OA-Type	1	4	5
OT-Type	6	0	6
PPD-Type	2	26	28
PPT-Type	10	7	17
Total	19	37	56

Table S4 Non-ginsenosides tentatively identified between cultivated and wild ginseng.

Peaks	t_R (min)	ESI (-) measured (*m/z*)	Adduct	Neutral formula	Error (ppm)	Major MS^2 ions (100%)	Tentative identification	References
1[a]	1.8	341.1085	-H	$C_{12}H_{22}O_{11}$	-1.39	[341]: 341(12), 179(26), 161(8)	sucrose	doi:10.3390/molecules23112855
1[b]		533.1718	-H	$C_{19}H_{34}O_{17}$	-0.83	[533]: 191(100)	quinic acid derivative	doi.org/10.1016/j.indcrop.2014.10.068
2	2.4	191.0189	-H	$C_6H_8O_7$	-4.27	[191]: 191(22), 111(100)	citric acid	doi.10.3389/fphar.2022.920979
3	7.5	345.0816	-H	$C_{14}H_{18}O_{10}$	0.12	[345]: 345(90), 330(100), 183(55)	methyl gallate-glucoside	doi:10.3390/molecules24061053
4	8.0	203.0818	-H	$C_{11}H_{12}O_2N_2$	-2.42	[203]: 203(100), 159(26),116(79)	tryptophan	doi:10.3390/molecules24061053
5	8.9	355.067	-H	$C_{15}H_{16}O_{10}$	0.96	[355]: 191(16), 147(11), 85(100)	p-coumaroyl aldaric acid	doi.org/10.3390/foods10092058
6	11.1	385.0776	-H	$C_{16}H_{18}O_{11}$	0.95	[385]: 191(17), 147(10), 129(9), 85(100)	feruloylgalactaric acid	doi:10.3390/molecules25235674
7	12.6	353.088	-H	$C_{16}H_{18}O_9$	0.58	[353]: 191(100)	chlorogenic acid	doi.org/10.1016/j.indcrop.2014.10.068
8[a]	13.4	471.2087	-H, +HCOO	$C_{18}H_{34}O_{11}$	0.91	[471]: 425(100), 293(91), 161(26)	Hexyl 6-O-glucopyranosyl-glucopyranoside	doi: 10.3389/fphar.2018.01288
8[b]		385.078	-H	$C_{16}H_{18}O_{11}$	0.95	[385]: 209(9), 191(17), 85(100)	feruloylgalactaric acid	doi:10.3390/molecules25235674
9	14.0	425.1672	-H	$C_{17}H_{30}O_{12}$	1.86	[425]: 379(20), 191(8), 179(48), 161(12), 101(31), 89(100)	glucopyranosyl-methylbutanoyl-glucopyranoside	doi.org/10.5650/jos.59.49
10	14.3	385.1144	-H	$C_{17}H_{22}O_{10}$	0.94	[385]: 223(100), 208(18), 179(47), 164(36)	sinapic acid hexoside	doi.org/10.1016/j.jep.2022.115604
11	14.8	447.1513	-H, +HCOO	$C_{18}H_{26}O_{10}$	0.55	[447]: 401(13), 269(100), 161(42)	benzyl alcohol xylopyranosyl-(1-6)-glucopyranoside	doi.org/10.3390/molecules26071855
12	15.9	381.1767	-H	$C_{16}H_{30}O_{10}$	0.21	[381]: 249(100), 161(34), 101(39), 89(13)	everlastoside C	doi.org/10.1016/j.jpba.2013.01.026
13	16.4	337.0929	-H	$C_{16}H_{18}O_8$	0.03	[337]: 191(100), 173(12)	coumaroylquinic acid	doi.org/10.1002/jssc.201700134
14	18.2	503.1774	-H	$C_{22}H_{32}O_{13}$	0.77	[503]: 503(100), 341(18), 179(10), 161(43), 101(46),89(41)	isoconiferoside	doi.org/10.3390/molecules16086577
15	18.6	367.1034	-H	$C_{17}H_{20}O_9$	-0.02	[367]: 193(7), 191(100), 173(17)	feruloylquinic acid	doi:10.3390/ijms20030475
